# Intersectional perspectives on the employment rate in Supported Employment for people with psychiatric, neuropsychiatric, or intellectual disabilities: A scoping review

**DOI:** 10.3233/WOR-211155

**Published:** 2023-02-14

**Authors:** Ingrid Witte, Thomas Strandberg, Sarah Granberg, Johanna Gustafsson

**Affiliations:** a School of Health Sciences, Örebro University, Örebro, Sweden; b School of Law, Psychology and Social Work, Örebro University, Örebro, Sweden; c The Swedish Institute for Disability Research, Örebro University, Örebro, Sweden; d School of Health and Welfare, Dalarna University, Falun, Sweden; e Audiological Research Centre, Faculty of Medicine and Health, Örebro University, Örebro, Sweden; f Centre for the Study of Professions, Oslo Metropolitan University, Oslo, Norway

**Keywords:** “Employment, Supported”, disabilities, “mental disorders”, intersectionality, “vocational rehabilitation”

## Abstract

**BACKGROUND:**

Supported Employment (SE) has shown better results in the employment rate for persons with disabilities than other methods within vocational rehabilitation, but how SE affects the employment rate for subgroups in the interventions needs further attention.

**OBJECTIVE:**

To examine previous research regarding the influence of intersecting statuses on the employment rate in SE for people with psychiatric, neuropsychiatric, or intellectual disabilities according to type of diagnosis, sex, race/ethnicity, age, level of education and previous work history.

**METHODS:**

A systematic literature search was conducted in nine databases including peer-reviewed articles from 2000 to April 2021. Articles presenting the employment rate in SE interventions according to the intersecting statuses listed in the objective were included.

**RESULTS:**

The searches identified 3777 unique records, of which 53 articles were included in data extraction. In most of the included articles, intersecting statuses did not affect the employment rate for people in the SE interventions with psychiatric disabilities. Few studies have examined neuropsychiatric and intellectual disabilities. A majority of the studies subjected to full-text analysis were excluded due to a lack of reporting of the effects of intersecting statuses on the employment rate. The studies that reported on the effects of intersecting statuses on the employment rate often had small samples and lacked statistical power.

**CONCLUSIONS:**

Intersecting statuses do not appear to affect the employment rate for people receiving SE interventions, but systematic reviews with pooled samples need to be undertaken because of the low reporting rate and underpowered sample sizes in existing studies.

## 1 Introduction

According to the United Nations Agenda for Sustainable Development [1] and the United Nations Convention on the Rights of Persons with Disabilities [2], persons with disabilities have the same rights to work opportunities as the rest of the population, but people with different types of disabilities have fewer opportunities to attain competitive employment than the population at large [3], even though many persons with disabilities aspire to be employed [4, 5]. To diminish the disadvantages for people with disabilities in the labor market, a method called Supported Employment (SE) has been developed in recent decades. The method has achieved better results regarding the employment rate for people with disabilities than other methods within vocational rehabilitation [6, 7]. Although research shows the effectiveness of SE, some reviews [7, 8] also notice that subgroup analyses of SE interventions exploring how SE affects different groups of people, such as different age groups, different disability groups (apart from severe mental illness (SMI)) and people from various cultural and ethnic backgrounds, still need to be performed.

### 1.1 Supported Employment

SE started to be developed in the United States in the 1970 s [9] and builds on the principles that persons with severe disabilities receive individual support by locating an appropriate job in the open labor market, by intensive job-site training, and by permanent ongoing support. This support is provided by a qualified staff person [10]. Initially, SE was developed for persons with intellectual disabilities (IDs) but expanded to persons with other disabilities, such as autism spectrum disorders and psychiatric disorders [9].

The manual-based approach to SE, Individual Placement and Support (IPS), for people with SMI emphasizes client choice, rapid job finding, competitive jobs, integrated work settings and follow-along support services and de-emphasizes excluding clients, extensive initial assessments, and prevocational training [11]. IPS has demonstrated a better effect on the employment rate than traditional vocational rehabilitation in systematic reviews [6–8, 12–16]. IPS is more extensively investigated than standard SE. Nøkleby et al. [7] examined the effects of SE in their systematic review. The SE studies in the review had few participants, and the results of the studies were not statistically comparable. However, the trend was that the SE methodology got more people work than other methods, although the results were uncertain.

### 1.2 Intersectionality and the employment rate for people with disabilities

The concept of intersectionality was launched by Crenshaw in 1989 and is based on the idea that people have several individual statuses at the same time and that these statuses intersect in different ways [17]. Intersecting statuses such as gender, race/ethnicity, class, and age have been considered in studies of intersectionality, and in recent years, disability has received some attention as a status to be studied [18]. According to Sommo and Chaskes [19], there are several aspects that need to be considered when incorporating disability into a study of intersectionality. Such considerations concern the heterogeneity and (sometimes) instability of a disability over time. Despite these considerations, there is a need to examine the issues that people with disabilities encounter in their everyday lives that relate to intersecting statuses such as gender, race, and class.

As for intersecting statuses and employment rates for people with disabilities, sex is a significant predictor of employment. Women with disabilities are less likely to be employed than men with disabilities and persons without disabilities in all regions in the world [3]. Ethnicity is also a predictor, and in the United States, unemployment rates are higher for Hispanic, Black and Asian persons with disabilities than for White persons with disabilities [20]. Age also affects the employment rate for persons with disabilities with the employment gap between persons with disabilities and persons without disabilities over the age of 50 increasing [20, 21]. Class, often measured by socioeconomic status (SES), is also an important status to include when studying intersecting statuses and disability. SES is difficult to capture, but the level of education is frequently used as a proxy of SES [22] and is often included as an intersecting status in different types of studies (including SE/IPS). Level of education is, at least in OECD countries, a predictor of employment success [23]. In addition, when studying employment rates, previous work history may be important to include because work experience is generally seen as a predictor of employment success [24].

The type of diagnosis is not an intersecting status for disability per se, but different types of diagnoses seem to have a hierarchical structure depending on the perceived severity and affect the employment rate for people with different types of disabilities [25]. Consequently, it is important to not ignore the type of diagnosis when studying employment rates. In this study, interest was especially focused on persons with psychiatric, neuropsychiatric, and intellectual disabilities because SE is mostly given to these groups [9].

### 1.3 Intersecting statuses and SE

Although SE, and especially IPS, have achieved better results regarding employment rates for people with disabilities than other vocational rehabilitation methods, few reviews have examined how intersecting statuses in relation to disability impact the results. Hence, systematic reviews on SE and IPS have requested more subgroup analyses [7, 8]. In a literature review from 2007, Loveland et al. [26] found that older people, minorities (e.g., African Americans or Hispanic) and people who had less than a high school education were less likely to obtain employment through SE. In another literature review using data up to 2010, Kirsh [27] found mixed results from previous studies on how intersecting statuses influenced the outcomes of SE. Some of the included studies found that statuses such as male sex and younger age were positively correlated with employment outcomes while other studies did not find these correlations. The author did not discuss the reasons for these differences in the results. Kirsch [27] also found that at least a high school education and previous work history were beneficial for obtaining jobs. In a thematic review of three studies from 2014, Lim et al. [28] found that IPS was efficient for persons with schizophrenia and schizoaffective disorders in different age groups but in different ways depending on the course of the illness. The authors called for more studies that control for other characteristics such as gender and ethnicity to further establish evidence for IPS. In a recent systematic review [29], the vocational outcomes of IPS for subgroups of diagnoses were examined. From the pooled data of 6 studies, IPS, in comparison with service as usual (SAU), was efficient for persons with schizophrenia and bipolar disorders in obtaining competitive employment; however, for persons with depression, there were no statistically significant differences between IPS and SAU. The authors considered that the group of persons with depression might be underpowered.

Thus, there are few previous reviews of the influence of intersecting statuses on the employment rate in SE, and they are often out of date. Only one identified review, which only studied diagnoses and no other intersecting statuses, used a systematic approach. Moreover, the results from previous reviews are ambiguous and inconclusive. Consequently, there is a need to systematically review how intersecting statuses influence the employment rate in SE.

### 1.4 Objective

The objective was to examine what has been reported regarding the influence of intersecting statuses on the employment rate in SE for people with psychiatric, neuropsychiatric, or intellectual disabilities according to the following: (i) type of diagnosis, (ii) sex, (iii) race/ethnicity, (iv) age, (v) level of education and (vi) previous work history.

## 2 Methods

Before choosing what type of review to conduct, a systematic reading of the articles included in the current systematic review by Nøkleby et al. [7] was performed. The results from the reading revealed that very few articles included in the review reported the results of intersecting statuses at the outcome level according to intervention group. According to Munn et al. [30], scoping reviews can be useful when examining types of available evidence in a research field and as a precursor to a systematic review in order to avoid obtaining an “empty” systematic review with very few included articles. Consequently, a scoping review was considered the best option for this review. The scoping review was conducted according to the method outlined by Peters et al. [31, 32]. The study protocol for this scoping review can be retrieved from the corresponding author. For the reporting of this review, the Preferred Reporting Items for Systematic Reviews and Meta-Analyses extension for Scoping Reviews (PRISMA-ScR) guidelines for reporting scoping reviews [33] were followed.

### 2.1 Criteria for considering studies for this review

Based on the population, context, and concept as outlined by Peters et al. [32], the criteria for eligible studies were as follows:

– *Population:* People of working age with a psychiatric, neuropsychiatric, or intellectual disability in need of support to obtain work in the open labor market. Populations that consisted of already employed study participants were excluded, and populations with mixed target groups were excluded if the participants were mixed in the results section. The term mixed target groups was used strictly.
– *Concept:* Employment rate achieved as a result of the SE/IPS interventions in the included studies, related to any of the following: (i) type of diagnosis, (ii) sex, (iii) race/ethnicity, (iv) age, (v) level of education and (vi) previous work history. If the statuses were reported only at baseline demographics or in the intention to treat group and not at outcome level according to the intervention group (i.e., the SE intervention), the study was excluded.
– *Context:* SE/IPS interventions labeled as SE/IPS interventions by the authors of the different articles. Studies not labeled SE/IPS or only examining augmented SE/IPS were excluded.

In addition, only peer-reviewed, original articles with quantitative study designs written in English, Danish, Norwegian or Swedish were included. Any other publication type and gray literature were excluded, and articles older than the publication year 2000 were excluded to ensure that only articles reflecting the current SE/IPS practice were included.

### 2.2 Method for searching and assessment

With support from a university librarian, the first author performed electronic literature searches in December 2019 and additional updated searches in April 2021. Searches were performed in the PubMed, PsycInfo, Cinahl, Social Services Abstracts, Sociological Abstracts, Business Source Premier, Eric, Scopus, and Web of Science databases. Due to the interdisciplinary nature of SE, the databases were chosen because of their different scopes and subject areas. Two search blocks were constructed: search terms related to Supported Employment and search terms related to mental/intellectual disability. Adding another search block with terms related to the employment rate reduced the results considerably, and this search block was abandoned to avoid excluding important results where the employment rate was not mentioned in the title/abstract. Both thesauruses, where it was applicable, and free text searches were used. For the free text search of the search block mental/intellectual disability, the categorization of disorders in the DSM-V was used to organize the search terms. Older terms and synonyms were also added to the block. For the Social Services Abstracts, Sociological Abstracts and Business Source Premier databases, only the search block of Supported Employment was used due to the few results. The limits of the search were publication language according to the inclusion criteria. The search strategy for the search in PubMed is presented in Table 1.

**Table 1:** Search strategy for database search in PubMed

*Terms related to Supported Employment*	1	(“Employment, Supported”[Mesh] OR “Supported Employment” OR “Individual Placement and Support”)
*Terms related to disability or diagnosis*	2	(“Mental Disorders”[Mesh])
	3	“Mental disorder” OR “Mental disorders” OR “Mental illness” OR “Psychiatric disorders” OR “Psychiatric disorder” OR “Psychiatric illness” OR “Neurodevelopmental disorders” OR “Intellectual disability” OR “Intellectual disabilities” OR “Learning disability” OR “Learning disabilities” OR “Learning disorder” OR “Intellectual disorder” OR “Intellectual disorders” OR “Intellectual developmental disorder” OR “Mental retardation” OR “Cognitive disability” OR “Cognitive disabilities” OR “Cognitive impairment” OR “Communication disorders” OR “Language disorder” OR “Language disorders” OR “Social communication disorder” OR “Autism spectrum disorder” OR “Autism spectrum disorders” OR Asperger* OR Autistic OR ”Attention Deficit Disorder” OR “Attention Deficit Hyperactivity Disorder” OR ADHD OR “Specific learning disorder”
	4	Psychotic OR “Psychotic disorder” OR “Psychotic disorders” OR Psychoses OR Psychosis OR “Schizotypal disorder” OR “Delusional disorder” OR “Schizophreniform disorder” OR “Schizophrenia” OR “Schizoaffective disorder” OR “Catatonia” OR “Catatonic disorder” OR “Schizophrenia Spectrum”
	5	“Bipolar disorder” OR ”Bipolar disorders” OR ”Bipolar I disorder” OR “Bipolar II disorder” OR “Cyclothymic disorder” OR “Affective illness” OR “Affective disorder” OR “Affective disorders” OR ”Manic depressive”
	6	”Depressive disorder” OR ”Depressive disorders” OR ”Disruptive Mood Dysregulation Disorder” OR ”Major Depressive Disorder” OR ”Persistent Depressive Disorder” OR Dysthymia OR Depression OR Melancholia
	7	“Anxiety Disorder” OR “Anxiety Disorders” OR Anxiety OR “Selective Mutism” OR “Social Anxiety Disorder” OR “Social Phobia” OR “Panic Disorder” OR “Panic Disorders” OR Agoraphobia OR ”Generalized Anxiety Disorder” OR GAD
	8	“Reactive Attachment Disorder” OR “Disinhibited Social Engagement Disorder” OR ”Posttraumatic Stress Disorder” OR PTSD OR ”Acute Stress Disorder” OR ”Adjustment Disorder” OR ”Adjustment Disorders”
	9	“Dissociative Identity Disorder” OR “Depersonalization Disorder” OR “Dissociative Disorder” OR ”Dissociative Disorders”
	10	“Somatic Symptom Disorder” OR ”Illness Anxiety Disorder” OR ”Illness Anxiety” OR ”Conversion Disorder” OR ”Conversion Disorders” OR ”Factitious Disorder” OR ”Factitious Disorders” OR “Somatoform disorder” OR ”Somatoform disorders”
	11	“Anorexia Nervosa” OR ”Bulimia Nervosa” OR ”Eating Disorder” OR ”Eating Disorders”
	12	”Insomnia Disorder” OR Insomnia OR ”Hypersomnolence Disorder” OR Hypersomnia OR Narcolepsy OR ”Sleep-Wake disorder” OR ”Sleep-Wake disorders”
	13	“Intermittent Explosive Disorder” OR “Conduct Disorder” OR “Conduct Disorders” OR “Antisocial Personality Disorder” OR ”Antisocial Personality Disorders”
	14	”Neurocognitive Domains” OR Delirium OR ”Neurocognitive Disorder” OR ”Neurocognitive Disorders”
	15	“Personality Disorder” OR “Personality Disorders” OR “General Personality Disorder” OR “Cluster A Personality Disorders” OR “Paranoid Personality Disorder” OR “Schizoid Personality Disorder” OR “Schizotypal Personality Disorder” OR “Cluster B Personality Disorders” OR “Borderline Personality Disorder” OR “Emotionally Unstable Personality Disorder” OR “Histrionic Personality Disorder” OR “Narcissistic Personality Disorder” OR “Cluster C Personality Disorders” OR “Avoidant Personality Disorder” OR “Dependent Personality Disorder” OR “Obsessive-Compulsive Personality Disorder”
	16	2 OR 3 OR 4 OR 5 OR 6 OR 7 OR 8 OR 9 OR 10 OR 11 OR 12 OR 13 OR 14 OR 15
	17	1 AND 16

After the initial database searches, duplicate articles were removed, and the first, second and fourth authors independently screened the titles and abstracts of the remaining articles according to the inclusion criteria. The articles were marked with yes, no or maybe for inclusion using the Rayyan software [34]. To ensure the reliability of the screening process, all titles/abstracts were screened by at least two reviewers. To eliminate cases of conflict or uncertainty regarding inclusion, the authors made decisions according to a consensus after screening. The full text review of the remaining articles was conducted by using the same procedure as for the title/abstract screening. The reference lists of all included articles were then searched manually to identify additional articles that might match the inclusion criteria. Articles not included in the Rayyan material were read in full text and assessed for eligibility using the inclusion criteria.

Data from eligible studies were charted using a data extraction form developed by the authors for this study. The form contained background information of all eligible studies (authors, year and journal of publication, country, aim/objective, study design, type of SE intervention and population) and study information on the overall employment rate in the SE intervention and the employment rate according to type of diagnosis, sex, race/ethnicity, age, level of education and previous work history. All authors extracted data independently and ensured that the data from each article were extracted by two reviewers. After the data extraction, the first, second and fourth authors jointly checked the results of the data extraction for errors.

Data synthesis was conducted by using descriptive statistics (frequency counts) of the variables in the data extraction chart. The average (unweighted arithmetic mean) employment rate for all the included articles which reported the employment rate at outcome was calculated and the differences in proportions of men and women in the SE/IPS-interventions were tested for statistical significance using 1-sample proportions tests with continuity correction with R [35].

## 3 Results

### 3.1 Background information of included articles

**Fig. 1:**
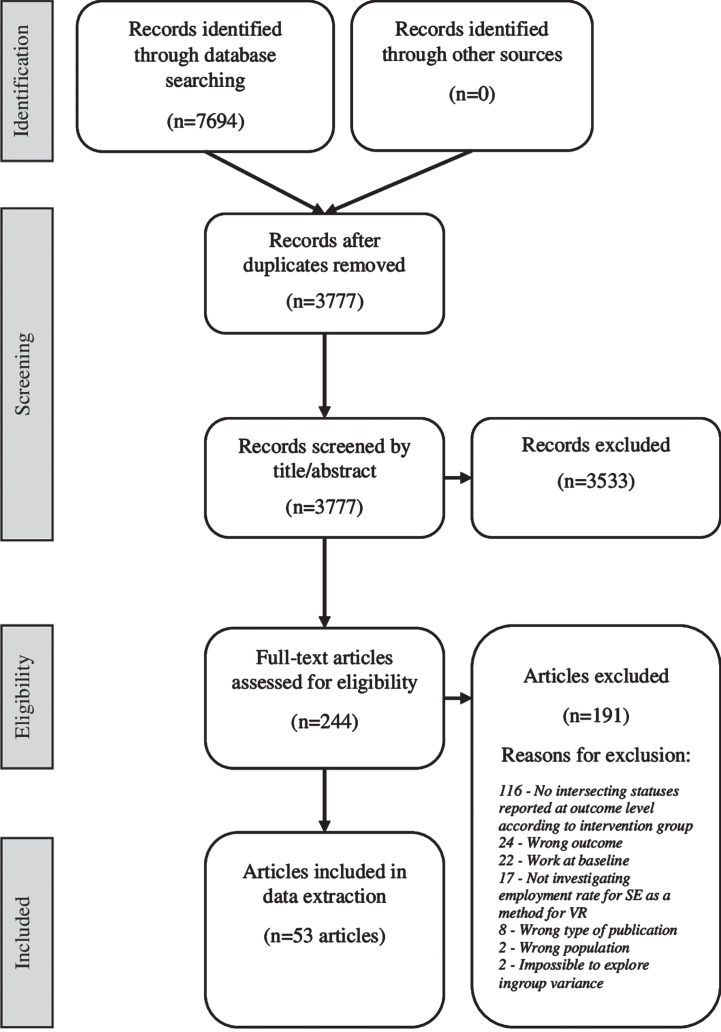
Flowchart of the review process adopted from the PRISMA flowchart by Moher et al. [36].

Out of 244 articles that had their full text analyzed, 116 were excluded because they did not report any intersecting statuses for employment rate at the outcome level according to the intervention group. Fifty-three articles met the inclusion criteria and were included in the data charting (Fig. 1). The background information of the 53 articles is given in Table 2. The 53 articles represent 46 unique study populations because some populations occur in several articles.

**Table 2:** Background information of the included articles

Ref. nr.	Author(s)	Year	Country	Intervention	Study design	Sample size	Population	Employment rate^a^	Studying intersecting statuses of:					
									Diagnosis	Sex	Race/ ethnicity	Age	Education	Work history
[37]	Barreira et al.	2010	The U.S.	SE	Register	99	Psychiatric disabilities	27/99, 27%	Yes	Yes	No	Yes	No	No
[38]	Becker et al.	2001	The U.S.	IPS	Experimental – CCT	73	Psychiatric disabilities	35/73, 47.9%	No	No	No	No	No	Yes
[39]	Beimers et al.	2010	The U.S.	SE	Observational	113	Psychiatric disabilities	53/113, 46.9%	Yes	Not reported	Yes	Not reported	Not reported	Not reported
[40]	Bond et al.	2016	The U.S.	IPS	Secondary analysis	49	Psychiatric disabilities	40/49, 81.6%	No	No	No	Yes	No	No
[41]	Browne et al.	2010	New Zealand	IPS	Register	49	Psychiatric disabilities	69.4%	Yes	No	Yes	Yes	No	No
[42]	Browne et al.	2009	New Zealand	IPS	Register	123	Psychiatric disabilities	64.2%	Yes	No	Yes	Yes	No	No
[43]	Burke-Miller et al.	2012	The U.S.	SE	Secondary analysis	649	Psychiatric disabilities	49.7%	Yes	Yes	Yes	Yes	Yes	Yes
[44]^b^	Campbell et al.	2010	The U.S.	IPS	Secondary analysis	307	Psychiatric disabilities	216/307, 70.4%	Yes	Yes	Yes	Yes	Yes	Yes
[45]^b^	Campbell et al.	2011	The U.S.	IPS	Secondary analysis	307	Psychiatric disabilities	216/307, 70.4%	Yes	Yes	Yes	Yes	Yes	Yes
[46]	Chang et al.	2016	Australia	IPS	Observational	60	Psychiatric disabilities	38/60, 63.3%	Yes	Yes	Yes	Yes	Yes	No
[47]^c^	Cook et al.	2008	The U.S.	SE/IPS	Experimental – RCT	648	Psychiatric disabilities+comorbidities	39%	Yes	No	No	No	No	No
[48]^c^	Cook et al.	2007	The U.S.	SE/IPS	Experimental – RCT	650	Psychiatric disabilities+comorbidities	39%	Yes	No	No	No	No	No
[49]	Fortin et al.	2017	Canada	SE	Observational	82	Psychiatric disabilities	44/82, 53.7%	Yes	Yes	Yes	Yes	Yes	Yes
[50]	Frounfelker et al.	2011	The U.S.	IPS	Observational	154	Psychiatric disabilities	48/154 31%	No	Yes	No	No	Yes	No
[51]^d^	Fyhn et al.	2020	Norway	IPS	Experimental – RCT	184	Psychiatric disabilities	N.A.^h^	No	No	No	Yes	Yes	No
[52]	Glynn et al.	2017	The U.S.	IPS	Experimental – RCT	56	Psychiatric disabilities	39/56, 70%	No	Yes	Yes	Yes	Yes	No
[53]	Gold et al.	2016	The U.S.	SE	Secondary analysis	167	Psychiatric disabilities	88/167, 53%	Yes	Yes	Yes	Yes	Yes	Yes
[54]	Henry et al.	2014	The U.S.	IPS	Register	3474	Psychiatric disabilities	1776/3474 51%	Yes	Yes	Yes	Yes	Yes	No
[55]	Hilarión et al.	2020	Spain	IPS	Observational	1620	Psychiatric disabilities	43%	Yes	Yes	No	Yes	No	No
[56]^d^	Holmås et al.	2021	Norway	IPS	Experimental – RCT	184	Psychiatric disabilities	N.A. ^h^	Yes	Yes	No	Yes	Yes	No
[57]	Howard et al.	2010	The U.K.	IPS	Experimental – RCT	109	Psychiatric disabilities	13/98, 13%	Yes	Yes	Yes	Yes	No	No
[58]	Jagannathan et al.	2020	India	SE	Observational	63	Psychiatric disabilities	32/63, 50.8%	No	Yes	No	No	No	No
[59]	Jones et al.	2001	The U.S.	SE/IPS	Observational	907	Psychiatric disabilities	580/907, 64%	Yes	Yes	No	Yes	No	Yes
[60]	Juurlink et al.	2019	The Netherlands	IPS	Secondary analysis	69	Psychiatric disabilities	31/69, 45%	Yes	No	No	No	No	No
[61]	Lucca et al.	2004	The U.S.	IPS	Register	90	Psychiatric disabilities	74/90, 82%	Yes	Yes	Yes	Yes	Yes	Yes
[62]	Macias et al.	2008	The U.S.	SE	Secondary analysis	174	Psychiatric disabilities	79/174, 45%	No	No	No	Yes	No	No
[63]	Mahmood et al.	2019	The U.S.	IPS	Experimental – single case	153	Psychiatric disabilities	72/153, 47%	Yes	Yes	Yes	Yes	Yes	Yes
[64]^e^	Metcalfe et al.	2017	The U.S.	IPS	Secondary analysis	1004	Psychiatric disabilities	522/1004, 52%	Yes	Yes	Yes	Yes	No	Yes
[65]^e^	Metcalfe et al.	2018	The U.S.	IPS	Secondary analysis	1004	Psychiatric disabilities	522/1004, 52%	Yes	Yes	Yes	Yes	Yes	Yes
[66]^f^	Mueser et al.	2014	The U.S.	IPS	Secondary analysis	67	Psychiatric disabilities	74%	No	No	Yes	No	No	No
[67]^f^	Mueser et al.	2004	The U.S.	IPS	Experimental – RCT	68	Psychiatric disabilities	74%	Yes	Yes	Yes	No	Yes	Yes
[68]^f^	Mueser et al.	2004	The U.S.	IPS	Experimental – RCT	68	Psychiatric disabilities	74%	Yes	No	No	No	No	No
[69]	Nygren et al.	2013	Sweden	IPS	Observational	65	Psychiatric disabilities	N.A. ^h^	Yes	Yes	No	Yes	Yes	Yes
[70]	Pelizza et al.	2019	Italy	IPS	Experimental – single case	54	Psychiatric disabilities	22/54, 40.7%	Yes	Yes	No	Yes	Yes	Yes
[71]	Pelizza et al.	2020	Italy	IPS	Observational	95	Psychiatric disabilities	39/95, 41.1%	Yes	Yes	Yes	Yes	Yes	Yes
[72]	Perkins et al.	2021	The U.K.	IPS	Register	779	Psychiatric disabilities	34.7% (1-year follow-up)	No	No	Yes	No	No	No
[73]	Petrakis et al.	2019	Australia	IPS	Register	136	Psychiatric disabilities	63/136, 46.3%	Yes	Yes	Yes	Yes	Yes	No
[74]	Reddy and Kern	2014	The U.S.	IPS	Secondary analysis	70	Psychiatric disabilities	15/70, 21%	No	No	No	Yes	No	No
[75]	Reddy et al.	2016	The U.S.	SE	Experimental – single case	65	Psychiatric disabilities	23/65, 35%	No	Yes	Yes	Yes	Yes	No
[76]	Rose et al.	2005	The U.K.	SE	Register	200	Intellectual disabilities	98/200, 49%	No	Yes	Yes	Yes	Yes	Yes
[77]	Rössler et al.	2019	Switzerland	IPS	Experimental – RCT	116	Psychiatric disabilities	67/116, 57.8%	Yes	Yes	No	Yes	Yes	Yes
[78]	Schaller and Yang	2005	The U.S.	SE	Register	365	Autism spectrum disorders+comorbidities	275/365, 75.3%	Yes	Yes	Yes	Yes	Yes	No
[79]	Schneider et al.	2009	The U.K.	SE	Observational	109	Psychiatric disabilities	32/109, 29%	Yes	Yes	Yes	No	Yes	No
[80]	Sherring et al.	2010	Australia	IPS	Experimental – single case	43	Psychiatric disabilities	33/43, 76.7%	No	Yes	No	Yes	Yes	Yes
[81]	Taylor and Bond	2014	The U.S.	IPS	Register	N.A.	Psychiatric disabilities	32%	Yes	Yes	Yes	Yes	Yes	No
[82]	Tuckerman et al.	2012	Australia	SE	Register	6244	Psychiatric, neuropsychiatric (autism) and intellectual disabilities	2565/6244, 41.1%	Yes	No	No	No	No	No
[83]	Twamley et al.	2012	The U.S.	IPS	Experimental – RCT	30	Psychiatric disabilities	56.7%	Yes	Yes	Yes	Yes	Yes	Yes
[84]^g^	Waynor et al.	2016	The U.S.	SE	Observational	105	Psychiatric disabilities	31/82, 38% (23 lost to follow-up)	No	No	No	No	No	Yes
[85]^g^	Waynor et al.	2018	The U.S.	SE	Observational	105	Psychiatric disabilities	31/82, 38% (23 lost to follow-up)	No	No	No	No	Yes	Yes
[86]	Wong et al.	2000	Hong Kong	SE/IPS	Observational	458	Psychiatric disabilities	308/458, 67.3%	Yes	Yes	No	Yes	Yes	No
[87]	Wong et al.	2004	Hong Kong	SE	Observational	748	Psychiatric disabilities	458/748, 61.2%	Yes	Yes	No	Yes	Yes	No
[88]	Wong et al.	2001	Hong Kong	SE	Observational	388	Psychiatric disabilities	267/388, 68.8%	Yes	Yes	No	Yes	Yes	No
[89]	Yamaguchi et al.	2020	Japan	SE	Observational	51	Psychiatric disabilities	26/51, 51%	No	Yes	No	Yes	Yes	Yes

^a^Actual numbers provided where available. ^b^Articles [44] and [45] represent the same population. ^c^Articles [47] and [48] represent the same population. ^d^Articles [51] and [56] represent the same population. ^e^Articles [64] and [65] represent the same population. ^f^Articles [66], [67] and [68] represent the same population. ^g^Articles [84] and [85] represent the same population. ^h^Did not report the employment rate for the entire study sample but examined intersecting statuses in relation to the employment rate.

Of the 46 populations studied, 34 originated from the Anglo-Saxon world. The objectives of the articles were related to examining the influence of one or several individual factors of the outcomes in the SE interventions in 34 cases [39–41, 43–45, 47–51, 54, 56, 59, 60, 62–70, 72, 74–78, 82–85]. In 19 cases, the objectives focused on other aspects (e.g., the effectiveness of an SE intervention) [37, 38, 42, 46, 52, 53, 55–58, 61, 71, 73, 79–81, 86–89].

As demonstrated in Tables 2 and 3, 31 of 46 studies had small sample sizes: less than 200 participants. Of these, 19 had samples with less than 100 participants. A total of 89% (41 of 46 studies) of the study populations consisted exclusively of persons with different types of psychiatric disabilities. Few articles examined SE for persons with neuropsychiatric disabilities or IDs (5 studies). Of the 38 studies reporting on sex distribution, 21 had a significantly higher proportion of men than women in the study sample. No study had a significantly higher proportion of women included. Of all the studies, 61% (28/46) did not report previous work history for their study participants. The studies reporting previous work history did so in several different ways.

**Table 3:** The populations of the included articles (based on 46 different populations)

Study sample		Studies n = 46 (%)	Article references (n = 53)
information
Sample size	<100 participants	19 (41)	[35, 36, 38, 39, 44, 47, 50, 56, 58, 59, 64–69, 72, 73, 78, 81, 87]
	100–199 participants	12 (26)	[37, 40, 48, 49, 51, 54, 55, 60, 61, 71, 75, 77, 82, 83]
	200–499 participants	5 (11)	[42, 43, 74, 76, 84, 86]
	500–999 participants	5 (11)	[41, 45, 46, 57, 70, 85]
	1000–9999 participants	4 (9)	[52, 53, 62, 63, 80]
	Unknown no. of participants	1 (2)	[79]
Diagnosis	Psychiatric disabilities	41 (89)	[35–44, 47–66, 68–73, 75, 77–79, 81–87]
	Intellectual disabilities	1 (2)	[74]
	SMI with some comorbidities with autism and ID	1 (2)	[45, 46]
	Psychiatric disabilities and neuropsychiatric disabilities	1 (2)	[67]
	Autism with comorbidities ID and MI	1 (2)	[76]
	Psychiatric disabilities, autism, ID	1 (2)	[80]
Sex	Reporting sex	38 (83)	[35, 37–40, 42–44, 47–49, 51–57, 59, 61, 64–79, 81–87]
	Did not report sex for the SE intervention	8 (17)	[36, 41, 45, 46, 50, 58, 60, 62, 63, 80]
Sex distribution	Equal sex distribution*	17 (45)	[35, 39, 40, 42–44^a^, 47, 49, 51, 54, 61, 64–70, 75, 77, 82, 83]
(of 38 reporting)	Nonequal sex distribution, more men than women*	21 (55)	[37, 38, 48, 52, 53, 55–57, 59, 71–74, 76, 78, 79^b^, 81, 84–87]
	Nonequal sex distribution, more women than men*	0
*Age*	Reporting mean age with SD and/or range	31 (67)	[35, 38–40, 42, 43, 47–49, 51, 52, 54–57, 59, 61, 64–66, 68–70, 72, 73, 75, 76, 78, 79, 81–87]
	Reporting age groups	5 (11)	[37, 41, 44, 67, 71]
	Reporting mean age without SD or range	2 (4)	[53, 74]
	Did not report age	8 (17)	[36, 45, 46, 50, 58, 60, 62, 63, 77, 80]
Mean age distribution (of 31 reporting)	Mean age < 30 yr. with SD < 5.9, range 16-39	4 (13)	[38, 39, 68, 76, 78]
	Mean age < 30 yr. with SD 7.23, range 18-64	1 (3)	[76]
	Mean age 32.7–49.9 yr., SD 7.3–16.8 range 16–69	24 (77)	[35, 40, 42, 43, 47–49, 51, 52, 54–57, 59, 61, 65, 69, 70, 72, 73, 75, 82, 84–87]
	Mean age 42 with SD 4	1 (3)	[79]
	Mean age > 50.3 yr. with SD 3.47, range > 45	1 (3)	[81]
Ethnicity/race	Reporting ethnicity/race	26 (57)	[35, 36, 38–40, 42, 43, 48, 51, 52, 55, 57, 59–61, 64–66, 68–70, 72–74, 76, 77, 79, 81–83]
	Reporting language	3 (7)	[44^c^, 47, 78]
	Reporting country of birth	2 (4)	[44^c^, 71]
	Did not report any of the above	16 (35)	[37, 41, 45, 46, 49, 50, 53, 54, 56, 58, 62, 63, 67, 75, 80, 84–87]
Education	>50% at least a secondary education	15 (33)	[37, 38, 42–44, 51, 52, (64–66)^d^, 67, 78, 79, 82–87]
	>50% less than a secondary education	2 (4)	[49, 54, 71]
	Mean years of education > 12 yr.	8 (17)	[48, 56, 61, 68, 69, 72, 73, 81]
	Mean years of education < 12 yr.	1 (3)	[75]
	10–12 years of completed education	1 (3)	[76]
	At least some postsecondary education	2 (4)	[59, 77]
	Did not report level of education	17 (37)	[35, 36, 39–41, 45–47, 50, 53, 55, 57, 58, 60, 62, 63, 70, 74, 80]
Work history	>50% worked competitively during the last 5 years	2 (4)	[51, 55]
	<50% worked competitively during the last 5 years	2 (4)	[36, 64–66]
	>50% any previous work experience	2 (4)	[56, 69]
	<50% any previous work experience	3 (7)	[37, 67, 68]
	Other ways of reporting previous work history	9 (20)	[38, 42, 43, 61, 74, 75, 78, 81–83, 87]
	Did not report previous work history	28 (61)	[35, 39–41, 44–50, 52–54, 57–60, 62, 63, 70–73, 76, 77, 79, 80, 84–86]

*As calculated with a 1-sample proportions test with continuity correction with R [88]. ^a^Strong tendency of more men than women, ^b^Based on average caseload for employment specialists, ^c^Nr. 44 reported both country of birth and language, ^d^50% >high school graduate, and 50% <high school graduate.

### 3.2 Employment rate and the influence of intersecting statuses on the employment rate

The measurement of the employment rate varied across the studies. Most studies measured the employment rate as obtaining a (competitive) job at any time during a follow-up period. These follow-up periods varied from 26 weeks up to more than 4 years, and 26 of 46 studies chose a follow-up period of 12 to 24 months. Three of the studies did not define the length of the follow-up period. Additionally, the length of time for employment to count as an employment outcome varied between the studies. Thirty-five of 46 studies did not define the length of employment at all (Table 4).

**Table 4:** Employment rate with definitions and the influence of intersecting statuses

		Studies (n = 46)	References to articles (n = 53)
Employment rate definitions	
Follow-up period	26 weeks	1	[74]
	6 months	5	[56, 82–86]
	12 months	14	[35, 37, 47, 48, 50, 52, 55, 70^a^, 72, 73, 76, 77, 81, 87]
	18 months	2	[38, 42, 43]
	24 months	10	[36, 39, 41, 45, 46, 51, 61–66, 75, 78]
	30 months	1	[58]
	36 months	1	[68]
	42–48 months	3	[40, 54^b^, 69]
	More than 4 years	4	[53, 59, 60, 71]
	15 months-6 years	1	[57]
	Employed at cross-section	2	[49^b^, 79]
	Not defined	3	[44, 67, 80]
Length of employment	At least one day	5	[58, 69, 70, 75, 87]
	At least one week	4	[35, 51, 60, 73]
	At least one month	2	[55, 78]
	Not specified	35	[36–50, 52–54, 56, 57, 59, 61–68, 71, 72, 74, 76, 77, 79–86]
Influence of intersecting statuses on employment rate			
Diagnosis	Not measured	14	[36, 38, 39, 48, 50, 56, 60, 70, 72–74, 78, 82, 83, 87]
	Not significant	24	[35, 37, 41, 42^c^, 44, 47, 51, 52, 55, 57–59, 62, 63, 67, 69, 71, 75–77, 79, 81, 84–86]
	Significant	6	[43^c^, 45, 46, 49, 54, 61, 64–66, 68]
	No significance tested	3	[40, 53, 80]
Sex	Not measured	11	[36, 38–40, 45, 46, 58, 60, 70, 72, 80, 82, 83]
	Not significant	26	[41, 42^c^, 44, 47–50, 54, 55, 57, 59, 61–69, 71, 73–78, 81, 86, 87]
	Significant	6	[35, 43^c^, 52, 79, 84, 85]
	No significance tested	3	[51, 53, 56]
	Unclear if measured	1	[37]
Race/ethnicity	Not measured	22	[35, 36, 38, 45, 46, 48, 49, 53, 54, 56–58, 60, 67, 68, 72, 75, 78, 80, 82–87]
	Not significant	17	[42^c^, 44, 47, 50–52, 55, 59, 61, 64–66, 69–71, 73, 74, 77, 81]
	Significant	6	[37, 41, 43^c^, 62, 63, 76, 79]
	No significance tested	2	[39, 40]
Age	Not measured	11	[36, 39, 45, 46, 48, 56, 58, 64–66, 70, 77, 80, 82, 83]
	Not significant	26	[35, 42^c^, 44, 47, 49 ^b^–51, 55, 57, 59, 61–63, 67–69, 71, 72, 74–76, 78, 81, 84–87]
	Significant	7	[41, 43^c^, 52, 54^b^, 60, 73, 79]
	No significance tested	3	[38, 40, 53]
	Unclear if measured	1	[37]
Education	Not measured	16	[35, 36, 38–40, 45, 46, 53, 55–58, 60, 70, 72, 74, 80]
	Not significant	26	[41, 42^c^, 44, 47–49^b^, 50–52, 59, 61–69, 71, 73, 75–78, 81, 84, 86, 87]
	Significant	5	[43^c^, 54^b^, 79, 82, 83, 85]
	Unclear if measured	1	[37]
Work history	Not measured	25	[35, 38–40, 44–46, 48–50, 52–56, 58, 60, 70–73, 76, 77, 80, 84–86]
	Not significant	13	[41, 51, 57, 59, 64–69, 74, 75, 78, 82, 83, 87]
	Significant	7	[36, 42^c^, 43^c^, 47, 61–63, 81]
	Unclear if measured	2	[37, 79]

^a^Two follow-up periods, 6 months, and 12 months. ^b^Same population but different follow-up periods and different measurements of employment. ^c^Same population but two different articles show different results.

The variations in the definition of the employment rate make it difficult to compare the studies. However, the mean employment rate in the 44 of 46 studies that did report this number was 50.8% with a standard deviation of 16.9. The variation in the employment rate among the included studies was thus large.

The included studies reported on the influence of the intersecting statuses to varying degrees, and ethnicity/race and work history were the least reported. Of the studies that examined whether the intersecting statuses had a significant influence on the employment rate, 24 of 30 reported no significance for diagnosis, 26 of 32 reported no significance for sex, 17 of 23 reported no significance for race/ethnicity, 26 of 33 reported no significance for age, 26 of 31 reported no significance for level of education and 13 of 20 reported no significance for work history (Table 4).

Of the studies reporting significant differences in the employment rate due to sex, 5 of the 6 studies reported that men were more likely to obtain employment than women. The sixth study by Taylor and Bond [81] studied differences in the employment rate depending on the employment specialists’ caseload and found that the higher the percentage of men on the employment specialists’ caseload, the lower the employment rate of the caseload.

Of the studies reporting significant differences in the employment rate due to previous work history, the results supported the notion that having previous work history positively affected obtaining employment. Campbell et al. [44], Fortin et al. [49] and the studies on the same study sample by Metcalfe et al. [64, 65] reported that previous work history was a predictor of obtaining employment. Two studies [63, 83] found that less time since a person’s last job increased the chances of obtaining employment. However, Campbell et al. [45] (same study sample as [44] but different statistical methods) found that the effect size for IPS in obtaining employment was larger for people with no working history than for people with a working history.

The studies that reported significant differences in the employment rate because of different diagnoses showed no clear tendencies. Campbell et al. [45] reported that the effect size of participating in IPS was larger for persons with psychotic disorders than for persons with bipolar disorders, but Campbell et al. [44] did not report this difference when using the same study sample. Two articles by Cook et al. on the same study sample [47, 48] found that persons with schizophrenia, IDs or any comorbidity had a lower employment rate. Holmås et al. [56] reported that the effect of IPS was larger for persons with SMI than for persons with moderate mental illness. Mueser et al. [68] showed that persons with a diagnosis of PTSD in addition to another diagnosis of SMI were less likely to work than people without an additional diagnosis of PTSD. Pelizza et al. [70] found that persons with SMI (and not a personality disorder) were more likely to work.

The results were inconclusive for the studies reporting significant differences in the employment rate due to race/ethnicity. Beimers et al. [39] found that non-White participants had a lower probability of obtaining employment, and Campbell et al. [45] found that African Americans had a larger effect size than Caucasians who had, in turn, a larger effect size than Latinos. Burke-Miller et al. [43] also reported that Hispanic/Latino individuals had a lower probability of obtaining employment, but Metcalfe et al. [64, 65] reported that Hispanic/Latino individuals had a greater probability of obtaining employment. Schaller and Yang [78] found that African Americans had a lower probability of obtaining employment, and Taylor and Bond [81] found that a higher percentage of Caucasian participants on an employment specialist’s caseload was positively related to the employment rate.

Similarly, the results were inconclusive for the studies reporting significant differences in the employment rate due to age. Burke-Miller et al. [43], Henry et al. [54] and Reddy et al. [75] found that younger participants had a higher probability of obtaining employment; however, Campbell et al. [45] found that IPS had a larger effect size for persons over 45 years, and Macias et al. [62] found that the SE intervention named PACT was especially efficient for older participants. Taylor and Bond [81] reported that a higher proportion of older participants on an employment specialist’s caseload was positively related to the employment rate.

The results of the studies that reported significant differences in the employment rate due to level of education were also mixed. Taylor and Bond [81] and Waynor et al. [84, 85] found that at least a secondary education was positively related to a higher employment rate; however, Campbell et al. [45] and Holmås et al. [56] found that the effect size for IPS was larger for persons with less than a high school education, and Wong et al. [87] found that the employment rate for less educated persons was higher than that for more educated persons.

A few of the included studies also reported on how the statuses that intersected with disability also intersected with each other. Barreira et al. [37] found that the subgroup of participants who were male, younger than age 50 and in good health were more likely than other participants to obtain employment. Perkins et al. [72] found no differences in the employment rate for different ethnic groups participating in IPS depending on sex or age. Waynor et al. [85] found that educational level was a significant predictor of obtaining employment and that female participants had higher educational levels, but there were no such associations between either type of diagnosis (SMI) or ethnicity and educational level.

## 4 Discussion

### 4.1 Main findings

The objective of this study was to examine what has been reported regarding the influence of intersecting statuses on the employment rate in SE for people with psychiatric, neuropsychiatric, or intellectual disabilities. Although the studies in this review do not describe intersectionality or intersecting statuses, they *do* examine intersecting statuses, and at first glance, the overall results of this scoping review suggest that the intersecting statuses in most cases do not significantly impact the employment rate of SE/IPS interventions. This finding is positive for the SE/IPS methodology as the intersecting statuses examined, such as sex, race/ethnicity, and age, are shown to impact the employment rate for persons with different types of disabilities in settings other than SE [3, 20, 21], and education and previous work experience are predictors of employment success in the general population [23, 24].

### 4.2 Methodological challenges in included studies

There are, however, several concerns that require attention when interpreting the results. As shown in the results, approximately half of the studies that were analyzed in full text were excluded because they did not report the effects of intersecting statuses on the employment rate at the outcome level according to intervention group. According to Macias et al. [62], this matter can be problematic because a zero difference in the effectiveness on the total study population can mask differences between subgroups at the outcome level. Considering that so many studies did not report the effects of intersecting statuses for employment rate at the outcome level according to intervention group, the results of this review have to be interpreted with caution because there are many uncertainties. Another methodological challenge when interpreting the results is the definition of the employment rate, which varies considerably between the studies, thus making the results of the included studies difficult to compare. This problem was also noticed in previous reviews [14, 16]. A third methodological challenge is the sample sizes of the included studies. Approximately two-thirds of the included studies had a sample size of less than 200 participants, and most of these studies had fewer than 100 participants, making it difficult to perform subgroup analyses with sufficient statistical power. Campbell et al. [45] note that many single studies of IPS have sample sizes that are too small to perform subgroup analyses. This problem is highlighted in some of the included articles with small sample sizes in this review [50, 60]. Consequently, there might be real subgroup differences that these small sample sizes do not detect. For example, regarding sex and race/ethnicity, for the studies in this review that reported significant differences for sex and race/ethnicity in relation to the employment rate, all but one (for each sex and race/ethnicity) had a sample size exceeding 300 participants. To obtain better study power, a solution is to perform systematic reviews with pooled samples where subgroup samples from several studies are merged into one subgroup sample, as Hellström et al. [29] performed to examine the effectiveness of IPS for subgroups of diagnoses. In their meta-analysis of four RCTs, Campbell et al. [45] also concluded that they had sufficient power to examine the influence of single factors but insufficient power to examine more complex structures, e.g., African American men. Only a few studies in this review had examined interaction effects between different intersecting statuses, and the small sample sizes of included studies might be a reason for this. The lack of statistical power for performing subgroup analyses in many single studies of SE/IPS is crucial when seeking to perform and understand intersectional analyses.

### 4.3 Studies reporting intersectional influence on the employment rate

Even if a majority of the studies in this review did not find the intersecting statuses to significantly affect the employment rate, it could be of interest to further investigate the studies that did report significant differences in the employment rate. Campbell et al. [44] note that with 24 predictor variables, at least one of them will be significant at the 0.05 level just by chance, as occurred in their study; and many of the included studies in this current review had many predictor variables. This result implies that some of the significant results might well depend on pure chance. However, some patterns do seem to be noteworthy. In the studies reporting significant differences depending on sex, all but one [81] found that men had higher employment rates than women. This finding is in line with study results from other settings for persons with disabilities [3]. Two out of three studies conducted in Hong Kong [86, 87] reported significantly more employed men than employed women. The third Hong Kong study [88], which did not report significantly more employed men, was a precursor to Wong et al. [87] with fewer participants, thus supporting the idea that small sample sizes might mask real subgroup differences. Wong et al. [86] discussed the possibility that the jobs obtained in the SE interventions were jobs with high physical demands that, out of tradition, may be more suitable for men. Hence, in some settings, the types of jobs available for SE participants seem to be more accessible for men. The type of diagnosis was reported to be significant in six cases, but the results from the studies were inconclusive and did not point in any particular direction. As for the intersecting factors of race/ethnicity, age, and level of education, the results were in some cases in line with findings from other settings where race/ethnicity and older age affect employment outcomes for people with disabilities [20, 21] and where a higher level of education is a general predictor of employment success [23]. These studies were also in line with previous reviews on SE [26, 27]. However, there were also contradictory results for race/ethnicity [45, 65], age [45, 62, 81] and level of education [45, 56, 87]. Regarding level of education, a possible explanation for these contradicting results is that SE/IPS participants mostly obtain entry-level jobs that do not require a higher educational level [27, 86]. The results of this review on how previous work history affects the employment rate in SE/IPS are in all but one case [45] in line with the notion that previous work history is a predictor of employment success [24, 27]. Another explanation for the inconclusiveness of the results might be the different organizations of the welfare regimes in the different settings of the included studies. A systematic review by Metcalfe et al. [90] found that the effect of IPS is stronger in societies with a weaker employment protection legislation, weaker integration of persons with disabilities and less generous disability benefits. These kinds of social policy conditions might also affect how people with different kinds of intersecting statuses fare in obtaining competitive employment. For example, as we could see earlier, women in Hong Kong seem to be disadvantages to men in obtaining competitive employment in SE and an explanation to this might as well be that Hong Kong provides minimal support for families and relies on the market and families to provide key welfare functions and also that parental leave is not gender neutral and working hours are not regulated [91]. All these interactions between welfare regimes, intersecting statuses and vocational rehabilitation interventions need more attention in research.

Some articles that used the same study sample acquired different results in different articles. In the case of the two articles using a Norwegian study sample [51, 56], the differences in the results may be due to differences in the follow-up period and employment measurements. For the two articles studying a pooled sample of four RCTs [44, 45], the differences in the results seem to depend on different statistical measures, thus highlighting the importance of using appropriate statistical measures.

### 4.4 Additional findings

Another topic that needs some attention is which persons participate in SE/IPS interventions. Scientific studies might not be representative of the typical participants of an SE/IPS intervention in all “real-world” settings, but they might give an indication. The absolute majority of the studies in this review exclusively had participants with psychiatric disabilities. Concerning IPS-studies, this is not surprising because IPS is developed for persons with SMI [11]. However, SE can also be given to other groups of people, but these other groups have not been included in studies of SE to the same extent [7]. This current review confirms this finding. Given that SE was developed for persons with IDs [9], this situation seems slightly strange. The scientific evidence for the effectiveness of SE for persons with IDs or, for example, autism spectrum disorders (ASDs) is not as strong as the scientific evidence of IPS for persons with SMI, but evidence from recent reviews [92, 93] suggests that SE can be efficient for people with ASDs and IDs.

A majority of the studies in this review that reported on sex had significantly more men than women as participants. This finding is in line with the results of the systematic review by Nøkleby et al. [7]. One possible reason for this situation could be an unequal sex distribution in the prevalence of the most common mental illnesses in IPS and SE participants: schizophrenia, bipolar disorders, and major depression [29]. However, the evidence for this explanation is unclear. According to a review on the prevalence of schizophrenia [94], the prevalence of schizophrenia according to sex is uncertain. Regarding bipolar disorders, the sex distribution seems to be equal [95], and for major depression, the prevalence is twice as high in women than in men [95].

### 4.5 An intersectional interpretation of the results

Because many studies do not report the effects of intersecting statuses on the employment rate at the outcome level according to intervention group and those that do are often underpowered, it is difficult to conduct a robust intersectional analysis of the results as the analysis will be uncertain. The intersecting statuses chosen in this study are all statuses that usually affect employment outcomes [3, 20, 21, 23–25]. However, it seems, with the cautions noted above, that they do not affect SE/IPS interventions in most of the studies. One explanation for this situation, considering that far from everyone in the SE/IPS interventions do get jobs, is the common notion of disability. From the perspective of intersectionality, people stay in many statuses at the same time, e.g., being white, older, and a woman. These statuses intersect and influence each other, but the status of disability might behave differently [18]. According to Barnartt [18], disability seems to be the master status of a person with a disability, and other statuses play minor roles and thus do not have as strong influences as they do when people do not have a disability. This explanation could be of interest if it were not for the UN [3], for example, reporting that women with disabilities are less likely than men with disabilities to be employed. With this example in mind, women with disabilities seem to be at a double disadvantage because the overall employment rate for people with disabilities is lower than for the population as a whole [3]. This double disadvantage also seems to play a role in other intersecting statuses such as disability and race/ethnicity [20] or disability and age [20, 21]. Nevertheless, there might be a case in which the status of disability plays the master status and other statuses moderate the effect of disability. Therefore, given that the intersecting statuses studied in this review often do not affect SE/IPS interventions, what components in SE/IPS moderate the effects of other influencing statuses that can be seen in other settings? Campbell et al. [44] attribute the effect to the individualized support that characterizes SE/IPS, and qualitative research on IPS [96] supports the idea that it is the person-centered, time-unlimited support is the key to enable and maintain competitive employment, but further research on this topic is needed.

### 4.6 Study strengths and limitations

This scoping review was comprehensive with an extensive database search complemented by a manual search. The reporting of the review has also been transparent. However, there are some limitations to the methodology. The search strategy in the databases with two search blocks, of which one was related to the diagnoses specified in the methods section, might have resulted in the exclusion of studies with the same categories of diagnoses if the types of diagnoses were not specified in the title, abstract or keywords that were screened. However, because the manual search of the included articles did not detect any further articles, this risk seems to be low. Gray literature was not searched for further references, which might be a limitation because valuable studies that could only be found in gray literature were not included. Another limitation is the language skills of the authors. A majority of the included studies were of Anglo-Saxon origin, which might have skewed the results since other major languages were lacking.

## 5 Conclusions and directions for future research

Intersecting statuses do not appear to affect the employment rate for people in SE interventions in a majority of cases, at least not for people with psychiatric disabilities. However, many studies do not report the influence of intersecting statuses, and those who do are often underpowered. There is therefore a need for more systematic reviews with pooled samples to properly assess the influence of intersecting statuses on the employment rate. There might also be a need for constructing studies that focus on intersectionality and intersecting statuses to be able to determine the effects of intersecting statuses for people with disabilities. If the positive outcomes for SE/IPS that were found in this scoping review remain after further studies, there will be a great need to examine why SE/IPS does not reproduce the patterns from the overall society.

## Data Availability

The data and materials can be obtained from the corresponding author upon reasonable request.
